# Universal prevention of distress aimed at pregnant women: a systematic review and meta-analysis of psychological interventions

**DOI:** 10.1186/s12884-021-03752-2

**Published:** 2021-04-01

**Authors:** Marjolein Missler, Tara Donker, Roseriet Beijers, Marketa Ciharova, Charlotte Moyse, Ralph de Vries, Jaap Denissen, Annemieke van Straten

**Affiliations:** 1 Department of Clinical, Neuro and Developmental Psychology & Amsterdam Public Health Research Institute, Vrije Universiteit Amsterdam, Van der Boechorststraat 7, 1081 BT Amsterdam, The Netherlands; 2 Radboud University, Behavioural Science Institute, Montessorilaan 3, 6525 HR Nijmegen, The Netherlands; 3 Department of Psychology Laboratory for Biological and Personality Psychology, Albert-Ludwigs-University of Freiburg, Stefan-Meier-Straße 8, D-79104 Freiburg i.. Br, Germany; 4 Radboud University Medical Center, Donders Institute for Brain, Cognition & Behavior, Heyendaalseweg 135, 6525 AJ Nijmegen, The Netherlands; 5 Vrije Universiteit Amsterdam, Medical Library, De Boelelaan 1117, 1081 HV Amsterdam, The Netherlands; 6 Department of Developmental Psychology, Utrecht University, Heidelberglaan 1, 3584 CS Utrecht, The Netherlands

**Keywords:** Universal prevention, Pregnant women, Maternal distress, Psychological interventions

## Abstract

**Background:**

There is sufficient meta-analytic evidence that antenatal interventions for women at risk (selective prevention) or for women with severe psychological symptoms (indicated prevention) are effective in reducing postpartum distress. However, women without risk or severe psychological symptoms might also experience distress. This meta-analysis focused on the effectiveness of preventive psychological interventions offered to *universal* populations of pregnant women on symptoms of depression, anxiety, and general stress. Paternal and infant outcomes were also included.

**Method:**

We included 12 universal prevention studies in the meta-analysis, incorporating a total of 2559 pregnant women.

**Results:**

Overall, ten studies included depression as an outcome measure, five studies included stress, and four studies anxiety. There was a moderate effect of preventive interventions implemented during pregnancy on the combined measure of maternal distress (*d* = .52), on depressive symptoms (d = .50), and on stress (d = .52). The effect on anxiety (d = .30) was smaller. The effects were not associated with intervention timing, intervention type, intervention delivery mode, timing of post-test, and methodological quality. The number of studies including partner and/or infant outcomes was too low to assess their effectiveness.

**Conclusions:**

This meta-analysis suggests that universal prevention during pregnancy is effective on decreasing symptoms of maternal distress compared to routine care, at least with regard to depression. While promising, the results with regard to anxiety and stress are based on a considerably lower number of studies, and should thus be interpreted with caution. More research is needed on preventing other types of maternal distress beyond depression. Furthermore, there is a lack of research with regard to paternal distress. Also, given the large variety in interventions, more research is needed on which elements of universal prevention work. Finally, as maternal distress symptoms can affect infant development, it is important to investigate whether the positive effects of the preventive interventions extend from mother to infant.

**Systematic review registration number:**

International prospective register of systematic reviews (PROSPERO) registration number: CRD42018098861.

**Supplementary Information:**

The online version contains supplementary material available at 10.1186/s12884-021-03752-2.

## Background

For many women, the period surrounding childbirth is accompanied by distress. Indeed, the prevalence of postpartum maternal distress symptomatology ranges from 8 to 40% for depression [1–4] and 13–40% for anxiety [5, 6]. In turn, these types of distress have been related to problems in children’s emotional, behavioural, and cognitive development (e.g. [7–9]). Preventing maternal distress will thus enhance both maternal and child well-being and health. The aim of the current review was to systematically review the evidence on the effectiveness of preventive interventions on distress offered to pregnant women.

The focus in most prevention studies of postpartum distress has been on *indicated* (or secondary) and *selective* (or primary) prevention. Indicated prevention means that an intervention is focused on pregnant women who already display symptoms of a psychological disorder without fulfilling the criteria for a full-blown disorder (e.g. [10, 11]). Selective prevention is aimed at pregnant women at risk for developing a disorder, for example women with a history of psychopathology, pregnancy complications, adverse life events, or low social support (e.g. [12–16]). Previous reviews and meta-analyses have suggested that both indicated [17, 18], as well as selective prevention [19–21] during the perinatal period are effective for the prevention of depression symptomatology. Even though research indicated that anxiety disorders might be more prevalent than depressive disorders during the perinatal period [22], much less is known about the effects of indicated and selective prevention on other disorders or symptomatology beyond depression, such as anxiety and stress. Recent reviews indicated that the few studies that have been done were either not effective [23], or that the number of available studies was too low to be able to assess their effectiveness properly [21].

In contrast to indicated and selective prevention, *universal* prevention is aimed at all women regardless of their risk status or symptoms. Given the relatively high level of maternal distress symptomatology after birth that prenatal symptoms likely continue in postpartum symptoms [9], and that postpartum distress might affect sensitive parenting important for a whole range of child outcomes [24–27], a preventive approach aimed at all pregnant women might be valuable, for both mother and child. Moreover, it is important to intervene as early as possible, preferably before birth, since parental distress symptomatology can impact child development from birth onwards [28, 29]. However, little is known about the effectiveness of universal prevention of symptoms of depression, anxiety and stress during pregnancy [21, 23].

Therefore, the aim of the current study was to systematically review and meta-analyze the available evidence on the effectiveness of preventive interventions on symptoms of depression, anxiety, and stress offered to *universal* populations of pregnant women compared to routine care. Previous meta-analyses included, but did not systematically investigate and differentiate, universal preventive psychological interventions [20, 21, 30, 31]. Moreover, this review will be the first to also include partner and infant outcomes. The prevalence of fathers’ symptomatology is estimated to be about 10% for mild to moderate depression [32, 33] and/or anxiety disorders [34]. As both maternal and paternal distress symptoms can impact infant development [9, 35–37], it is important to investigate whether effects of universally applied psychological interventions extend from the parents to the infant.

## Method

### Protocol and registration

This meta-analysis has been prospectively registered at the international prospective register of systematic reviews: Prospero (https://www.crd.york.ac.uk/prospero/, ID: CRD42018098861).

### Information sources and search

A comprehensive literature search was performed in the bibliographic databases PubMed; Embase; Ebsco/PsycINFO; Ebsco/CINAHL; and Wiley/Cochrane Library in collaboration with a medical librarian. Databases were searched from inception up to 15 November 2018. The following terms were used (including synonyms and closely related words) as index terms or free-text words: “Parents”, “Pregnancy”, “Prevention”, “Education”, “Cognitive therapy”, “Stress”, “Anxiety”, “Depression”, “Well-being”, “RCTs”. The search was performed without date, language or publication status restriction. Duplicate articles were excluded. The full search strategies for all databases and the number of identified items per database can be found in Additional File 1.

### Eligibility criteria

The following eligibility criteria were applied during the data collection process: (a) randomized controlled trials; (b) testing psychological interventions for pregnant women (with or without inclusion of their partner); (c) starting prenatally; (d) aimed at preventing maternal depression, anxiety and/or stress (e) comparing the active condition with care-as-usual, placebo or waitlist and (f) published in English in (g) international peer-reviewed journals. Care-as-usual could consist of regular consults with professionals in (prenatal) health care, such as midwives, general practitioners, or obstetric nurses. These consults are typically focused on monitoring the health of the mother and the fetus and on providing information about pregnancy and the delivery. Except for the psychological character of the interventions, there were no specific criteria for eligibility. Examples of (elements of) interventions that could be included are: psychoeducation, relaxation techniques, mindfulness, and social support. The interventions could be implemented through education materials (booklets, websites, or videos), individual meetings, group meetings, home visits, or combinations of these. Trials were excluded if they were aimed at indicated prevention (pregnant women with pre-existing psychopathology following DSM-IV or scoring above cut-off on validated clinical measures such as the Edinburgh Postnatal Depression Scale (EPDS, [38]) or at selective prevention (aimed at pregnant women with a high risk to develop psychopathology such as low-income pregnant women, teenage pregnancies, or HIV positive pregnant women). Furthermore, studies reporting insufficient outcome data to calculate effect sizes were excluded (such as the non-reporting of standard deviations, or the reporting of plotted data only).

### Data collection process

After our literature search, we removed duplicates. Two independent assessors (MM and TD) examined the titles and abstracts. The full-text of all remaining potentially eligible papers was retrieved after which the selection of studies, based on the above eligibility criteria, was done by two researchers (MM for all studies and MC or CM for half of the studies). Differences between the two raters were solved by discussion. In case of disagreement, the paper was discussed with the other members of the review team (AvS and/or TD) until consensus was reached. For data extraction, a piloted standardized form was used. This form included the following categories: study characteristics, risk of bias assessment, and data to calculate effect sizes. Study characteristics that were coded are: 1) year of publication; (2) country (high/low income); 3) participant characteristics (*N*, age, SES), 4) inclusion of the partner (yes/no); 5) type of intervention (psychoeducation, cognitive-behavioural therapy (CBT), mindfulness, or another intervention); 6) timing of the intervention (prenatal or a mix of prenatal and postnatal implementation); 7) delivery method of the intervention (individual, group, or mixed format); 8) materials used (e.g. booklet or video); 9) number of sessions; 10) training and supervision of the providers of the intervention (type and frequency of training); 11) method of recruitment (ads, hospital, midwives, other); 12) type of control group and/ or characteristics of the alternative treatment (wait-list, care-as-usual, alternative intervention); 13) type of randomization and number of arms; and 14) primary and secondary outcomes of the study.

### Risk of bias in individual studies

Risk of bias was assessed with The Cochrane Risk of Bias Assessment Tool [39]. This tool consists of the following criteria: random sequence generation, allocation concealment, blinding of participants and personnel, blinding of outcome assessment, incomplete outcome data and selective reporting. Again, two researchers (MM, and MC or CM) independently assessed risk of bias for each study. Discrepancies in ratings between the two researchers were resolved by discussion, led by a third researcher (AvS or TD).

### Statistical analysis

We performed a random-effects meta-analysis, using the ‘Comprehensive Meta-analysis’ software package for Windows (CMA; version 3; available from www.metaanalysis.com). To calculate the pooled effect size of the intervention, we used the post-test measures of different measures of distress and expressed them in Cohen’s *d* [40]. This value refers to the number of standard deviations the intervention group scores better (or worse) than the control group. An effect size of 0.20 can be considered as small, of 0.50 as moderate, and 0.80 as large [40]. For studies using different instruments measuring the same outcome (i.e. two different depression scales), the outcomes were combined in one effect size per outcome (the mean of the two separate effect sizes). When multiple interventions were compared with a non-treated control group [41, 42], the effect of the intervention was compared to both the active intervention as well as to the control condition. Thus, in this case, we included both comparisons (intervention A – vs. control group and intervention B vs. control group) in our analysis.

First, we calculated the pooled effect size for studies measuring maternal distress, thereby combining depression, anxiety, stress, and/or parenting stress. We checked for outliers (defined as a case in which the 95% confidence interval of an individual study did not overlap with the 95% confidence interval of the overall pooled effect size). After removal of two outliers, we repeated the main analysis. We then repeated the analysis (without the two outliers) on the measures of distress separately, namely depression, anxiety, and (general or parenting) stress.

Statistical heterogeneity was assessed with the *I*^*2*^-statistic (fixed effects model), which refers to the variance between studies as a proportion of the total variance. High percentages indicate substantial heterogeneity. Numbers-needed-to-be-treated (NNT) were calculated from the effect sizes. Publication bias was examined by a visual inspection of the funnel plot and by Egger’s test of the intercept. An estimation of the effect size while taking publication bias into account was performed by means of the Duval and Tweedie trim and fill procedure.

Sub-group analyses on the combined outcome of depression, anxiety and stress, were performed for the following variables: timing of the intervention (prenatal only or a combination of prenatal and postpartum elements); intervention type (psychoeducation; CBT; mindfulness or other interventions); intervention delivery mode (delivered in a group or on an individual basis); whether the partner was included in the intervention; timing of post-test (during pregnancy or in the first 6 months after birth); and methodological quality (based on the risk of bias assessment performed through the Cochrane tool). We used the mixed effect model, which pools studies within subgroups with the random effects model but tests for significant differences between subgroups with the fixed effect model.

## Results

### Study selection

**Fig. 1:**
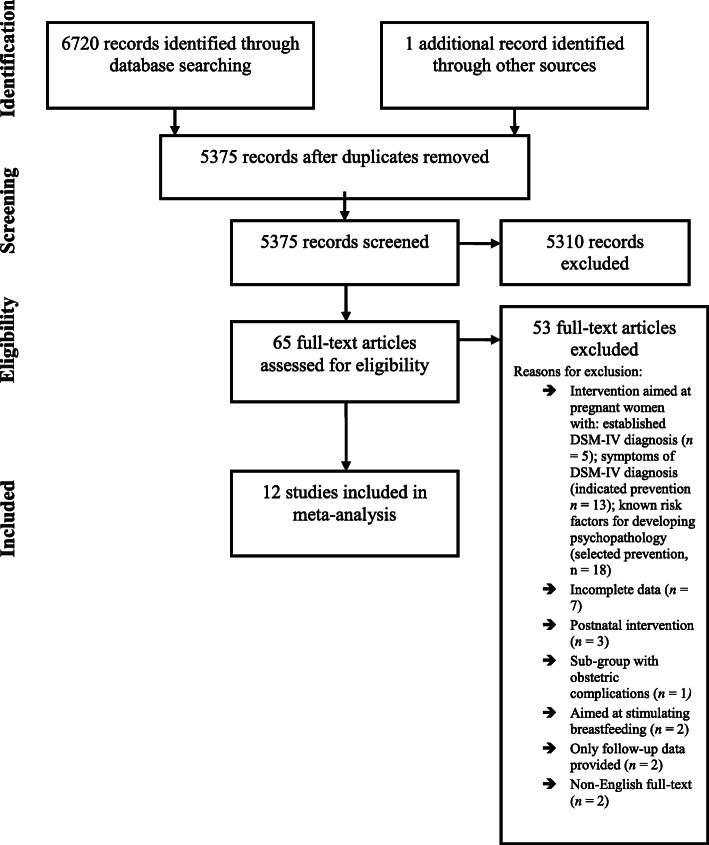
PRISMA flow chart of included studies

After removal of duplicates (*n* = 1345), titles and abstracts of 5375 references were screened for eligibility (Fig.1). Based on title and abstract, 5310 references were excluded at this stage. For 65 references, we retrieved the full-text. Based on the full-text information, 53 references were excluded, resulting in a final inclusion of 12 universal prevention studies in the meta-analysis (Table 1). These 12 studies incorporated a total of 2559 pregnant women. In 3 studies, the partner also participated (*n* = 360).

**Table 1:** Characteristics of 12 randomized controlled trials of psychological interventions in pregnant women

Author	Year	Country	Intervention type	Outcome(s)	Delivery method	Control	N_sess_	Inclusion of partner	Timing intervention	N_pat_	Risk of bias
Akbarzadeh	2016	Iran	Psychoeducation	Anxiety (STAI)	Group	CAU	4	Yes	Prenatal	126 couples	High
Beattie et al.	2017	Australia	Mindfulness	Stress (PSS-10); Depression (EPDS)	Group	CAU (Pregnancy Support Program)	8	No	Prenatal	48 women	Low
Daley-McCoy et al.	2015	England	Psychoeducation	Depression (EPDS)	Group	CAU	1	Yes	Prenatal	63 couples (70 women and 65 men)	Some concerns
Feinberg & Kan	2008	United States	CBT	Depression (CES-D); Anxiety (TMAS)	Group	CAU (+ child care brochure)	8	Yes	Mixed (4 postnatal sessions)	169 couples	Some concerns
Gao et al.	2010	China	IPT	Depression (EPDS; GHQ)	Group (prenatal)/ Individual (postpartum phone call)	CAU	3	No	Mixed (1 postnatal session)	194 women	Some concerns
Haga et al.	2019	Norway	Multimodal	Depression (EPDS)	Individual (10-min online self-help sessions)	CAU	44	No	Mixed (11 prenatal sessions)	1342 women	Some concerns
Khorsandi et al.	2016	Iran	CBT	Stress (PSS-14)	Group	WL	8	No	Prenatal	64 women	Some concerns
Mao et al.	2012	China	CBT	Depression (PHQ-9; EPDS)	Group/Individual (1 coaching session)	CAU	5	No	Prenatal	240 women	Low
Matvienko-Sikar & Dockray	2017	Ireland	Mindfulness	Stress (PDS); Depression (EPDS)	Self-help	CAU	11	No	Prenatal	46 women	High
Milgrom et al.	2011	Australia	Psychoeducation	Depression (BDI-II); Anxiety (DASS); Stress (DASS)	Self-help (workbook)/individual (phone)	CAU (+ intervention workbook after study)	8	No	Mixed (1 postnatal session)	143 women	Some concerns
Ramezani et al.	2017	Iran	CBT	Depression (Austin Inventory; EPDS)	Group	AI (solution-focused counselling) + CAU	4	No	Prenatal	85 women	High
Woolhouse et al.	2014	Australia	Mindfulness	Depression (CES-D; DASS); Anxiety (STAI; DASS); Stress (PSS; DASS)	Group	CAU	• 6	No	Prenatal	32 women	Some concerns

Abbreviations. *BDI* Beck Depression Inventory; depression; *CAU* care as usual; *CBT* cognitive behavioural therapy; *CES-D* Center for Epidemiological Studies, Depression Scale; *DASS* Depression Anxiety Stress scales short form; *EPDS* Edinburgh Postnatal Depression Scale; *HADS-D* Hospital Anxiety and Depression Scale; *GHQ* General Health Questionnaire; *LQ* Leverton Questionnaire; *IPT* Interpersonal psychotherapy; *N*_*pat*_ number of patients; *N*_*sess*_ number of sessions; *PDS* Prenatal Distress Scale; *PHQ-9* Patient Health Questionnaire; *POMS* Profile of Mood States (Depression / dejection scale); *PSS* Perceived Stress Scale; *SH* self-help; *STAI* State and Trait Anxiety Inventory; TMAS – Taylor Manifest Anxiety Scale; WL – waiting list.

### Study characteristics

The 12 included studies were published between 2008 and 2018. Overall, ten studies included depression as an outcome measure, five studies included stress, and four studies anxiety. Four of them focused exclusively on depression, whereas the other six studies also included anxiety and stress. Only two studies focused only on anxiety or stress. Two of the studies [41, 42] compared more than one intervention group with the control condition, resulting in 14 comparisons. In one study, [43] a newly developed mindfulness intervention was compared with a regular pregnancy support program, while in the other studies a non-treated control condition was used. Most studies (*n* = 9) were performed in high-income countries. In all studies pregnant women were recruited through antenatal clinics of local hospitals. In one of the studies [44] women were additionally recruited through private clinics and the use of advertisements (online and on paper). With regard to parity, in most of the included studies, only primiparous women were included (*n* = 7). In three of the remaining studies, the majority of women was expecting their first child. For the other two studies, about one third of the women was primiparous. Given the low number of studies including multiparous women, we decided not to perform subgroup analyses with regard to parity.

The studies we included aimed for universal recruitment. The majority of the studies (*n* = 9) used various indicators of the presence of psychopathology (current or former, diagnosis or symptomatology) as an exclusion criterion. The remaining three studies did not use any indicator of the presence of psychopathology (symptoms or diagnosis) as an explicit exclusion criterion. About 20 to 30% of women scored above a (varying) cut-off score indicating potential depression in three of the studies that did use (a diagnosis of) psychopathology as an exclusion criteria [44–46] and in two studies which did not use such an exclusion criterion [47, 48]. Overall, the baseline scores of all studies showed mean depression, anxiety, and stress scores that were well below clinical cut-off, except for the depression scores in one study [44]. In this study, participants showed relatively high baseline EPDS scores.

The 14 comparisons included the following interventions: psychoeducation (*n* = 3); cognitive-behavioral therapy (*n* = 4); mindfulness/relaxation (*n* = 4); interpersonal Psychotherapy (IPT; *n* = 1); solution-focused counseling (*n =* 1*)* and one study [48] used an extensive online multimodal intervention consisting of elements of e.g. CBT, mindfulness,, and meta-cognitive therapy. The interventions were implemented prenatally only (*n* = 10) or took place both before and after delivery (*n* = 4). The majority of the studies compared their intervention to routine care (*n* = 10), or a supportive intervention resembling routine care (*n* = 1). In two studies, an alternative intervention was also compared to a non-treated control group. Only one study used a wait-list control condition. The studies did not report how the intervention and control conditions were presented to the participating women, with one exception: in the Woolhouse et al. study [46] women were informed that they would participate in the evaluation of an intervention to support them in managing their stress levels.

The number of sessions ranged from 1 to 44. The study with 44 (10 min) sessions was an outlier with respect to the number of sessions [48]. The mean number of sessions with this study excluded was 9.08. This was also true for the number of participants (*n* = 1342) in the Haga et al. (2019) study [48], which was considerably higher than in the other studies (mean number of participants of the other studies was 110). Most interventions were implemented in a group (*n* = 9); or used a combined format with prenatal group sessions and additional individual care (e.g. one individual coaching session; *n* = 2) Three interventions were offered online in a self-help format. Two of these were unguided [44, 48] while in one intervention, participants were supported by phone [45]. Most interventions were provided by a professional in (mental) healthcare, namely a midwife (*n* = 4); a psychologist (*n* = 3); or an obstetrician (*n* = 1). In one study, group sessions were facilitated by a trained female-male team from childbirth education departments of local hospitals [49]. No information about the facilitators was provided in two studies [41, 50]. Most intervention facilitators received a specific training (*n* = 9) and to a lesser extent also supervision (*n* = 5) during the intervention.

### Risk of bias

Of the 12 included studies, four reported an adequate *random sequence generation*. For the remaining studies, the description of this procedure was not sufficient to judge this criterion. Importantly, no significant baseline differences emerged between the intervention and control groups in 11 studies, indicating adequate randomization. One study did not report baseline distress data [42]. In five studies, the *allocation procedure* was adequately concealed, while for six studies, this remained unclear. In one study, there seemed to be problems with the concealment of the random allocation process [44] because the generation of the allocation sequence, the enrollment of participants, and the random allocation were all done by the same researcher. Five studies were judged as low-risk on the *incomplete outcome data* criterion, either because of the use of an intention-to-treat analysis (*n* = 4) or the reporting of a low drop-out rate (defined as at least 80% of the participants completing the intervention and the post-intervention measurements). Conversely, because of relatively high drop-out rates (drop-out rate > 20%) and no clear reporting of the reasons for these high drop-out rates, five studies were judged as high risk. Two studies did not report enough information to judge this criterion. For the majority of studies (*n* = 9), we were unable to judge whether there was any *selective reporting*. The availability of a study protocol or trial registration justified a low-risk judgement for two studies only. One study was assessed as high risk. Concerning potential *other sources of bias*, only one study was judged as high-risk because of unclarities in the reporting of the study (mainly with regard to analysis methods). There were no clear indications for the presence of *researcher allegiance* in the included studies.

### Synthesis of results

#### Main analysis: effect of the interventions on maternal distress

The overall effect of preventive psychological interventions implemented during pregnancy on different measures of maternal distress (depression, anxiety, and stress) was considerable (Cohen’s *d* = .52; 95% CI .29 ~ .74; Table 2). The *I*^*2*^ statistic showed a large (and significant) percentage of heterogeneity of 76%. Two studies resulted in considerable higher effect sizes than the other included studies, and could thus be considered outliers [48, 51]. The removal of these two outliers resulted in a somewhat smaller effect size of *d* = .47 (95% CI .31 ~ 0.62) and a considerable decrease in the percentage of heterogeneity (I^2^ = 27%, non-significant). To limit this heterogeneity, we decided to perform all analyses without the two outlying studies. When looking at the distress outcomes separately, substantial effect sizes were obtained for depression (*n* = 10; *d* = .50; 95% CI .32 ~ .67), stress (*n* = 5; *d* = .52; 95% CI .28 ~ .75) and anxiety (*n* = 4; *d* = 0.30; 95% CI < 0.01 ~ 0.59). Heterogeneity was low in these analyses for the separate outcomes.

**Table 2:** Psychological interventions vs care-as-usual control groups for preventing distress among pregnant women: effect sizes^a^

	N _comp_	d	95% CI	*p*-value	I^2^	NNT
All studies	14	0.52	0.29 ~ 0.74		76.14*	3.50
2 outliers removed	12	0.47	0.31 ~ 0.62		26.56 ns	3.85
Lowest ES excluded^b^	12	0.50	0.36 ~ 0.64		14.9 ns	3.62
Highest ES excluded^b^	12	0.43	0.25 ~ 0.61		41.54 ns	4.20
Depression only^b^	10	0.50	0.32 ~ 0.67		26.99 ns	3.62
Anxiety only^b^	5	0.30	< 0.01 ~ 0.59		43.03 ns	5.95
Stress only^b^	5	0.52	0.28 ~ 0.75		00.00 ns	3.50
Timing intervention^b^				0.63		
• Prenatal	9	0.52	0.36 ~ 0.68		07.32 ns	3.50
• Mixed	3	0.37	0.16 ~ 0.58		60.95 ns	4.85
Intervention type^b^				0.54		
• Psychoeducation	3	0.34	0.03 ~ 0.65		22.77 ns	5.26
• CBT	3	0.43	0.24 ~ 0.62		66.23 ns	4.20
• Mindfulness	3	0.32	−.14 ~ 0.78		0.00 ns	5.56
• Other	3	0.62	0.39 ~ 0.84		43.81 ns	2.96
Delivery mode^b^				0.54		
• Group	10	0.45	0.32 ~ 0.58		34.52 ns	4.00
• Individual	2	0.61	0.13 ~ 1.10		00.00 ns	2.99
Inclusion of partner^b^				< 0.01		
• No	8	0.59	0.43 ~ 0.75		0.00 ns	3.09
• Yes	4	0.25	0.04 ~ 0.45		0.00 ns	7.14
Risk of bias^b^				0.40		
• High	5	0.55	0.32 ~ 0.78		38.08 ns	3.31
• Low	2	0.56	0.32 ~ 0.81		00.00 ns	3.25
• Medium	5	0.35	0.16 ~ 0.54		26.78 ns	5.10
Timing post-test^b^				0.93		
• Pregnancy	5	0.48	0.30 ~ 0.67		0.00 ns	3.76
• 0–6 m pp	7	0.45	0.27 ~ 0.62		51.54 ns	4.00

^a^ random effect models; ^b^ analysis did not include four outliers (Khorsandi; Haga); *N*_*comp*_ number of comparisons; ns: not statistically significant; NNT Numbers Needed to Treat

Some studies reported more than one effect size because they included more than one measure to report an outcome (e.g. two different depression measures). In these cases, we used a pooled effect size in the above analyses. However, we also conducted sensitivity analyses, in which we included only one comparison for each study. First, for each study, we only included the comparison with the largest effect size. Then, we analyzed only the comparison with the smallest effect size. The resulting overall effect sizes were comparable to the overall effect size (Table 2). However, when including only the comparisons with the highest effect size, the percentage of heterogeneity increased to a moderate level (though again non-significant).

#### Sub-group analyses

The sub-group analyses for intervention timing, intervention type, intervention delivery mode, timing of post-test, and methodological quality did not show statistically significant differences on the combined outcome of maternal distress (Table 2). Only one subgroup analysis produced a statistically significant difference: interventions which did not include the partner showed a larger effect on maternal distress (*d* = .59) than studies that did include the partner (*d* = .25; *p* < .01; Table 2).

#### Publication bias

**Fig. 2:**
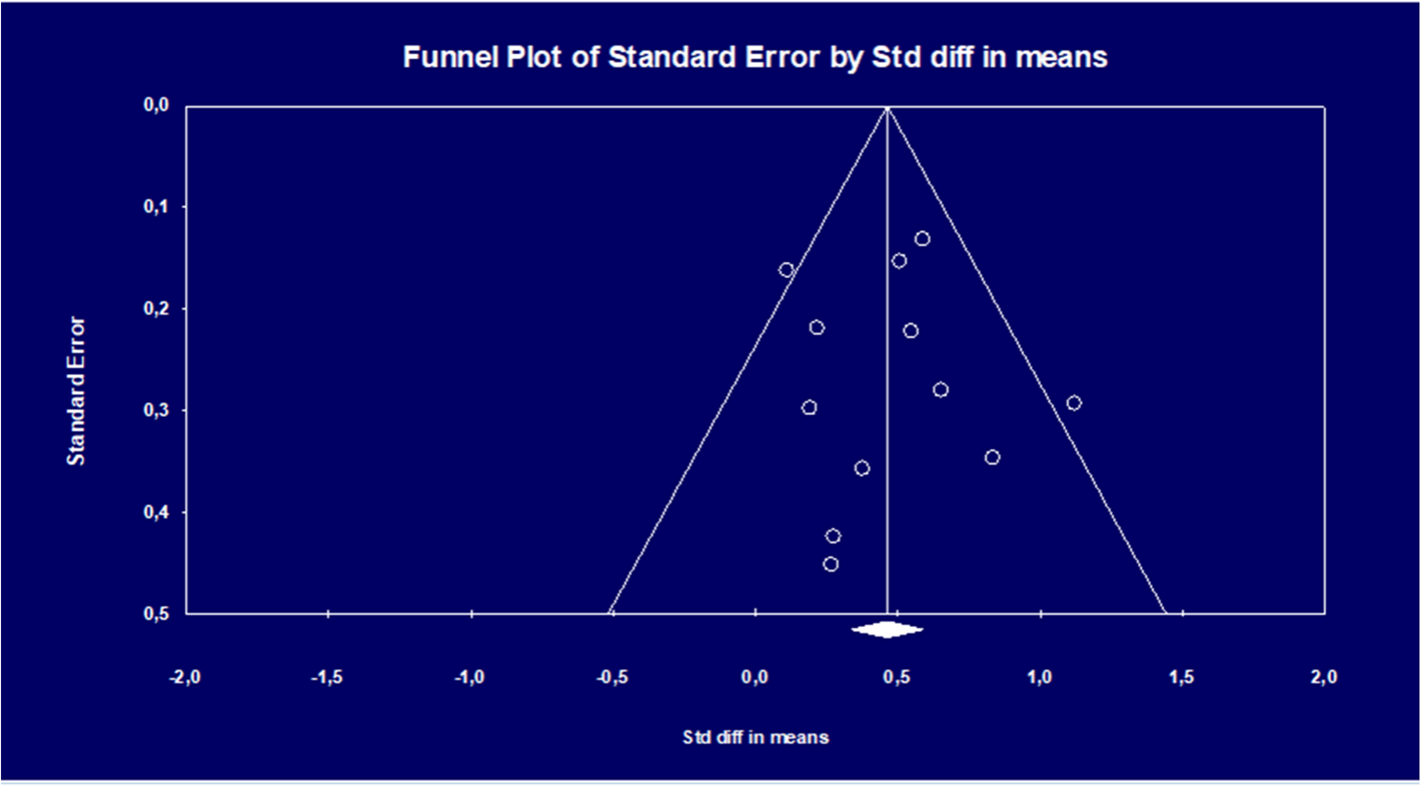
Funnel plot of standard error by effect size (Cohen’s *d*) in studies comparing preventive psychological interventions aimed at preventing maternal distress with non-treated control conditions in universal populations of pregnant women

We tested for publication bias on the maternal distress outcome data first by visually inspecting the funnel plot (Fig. 2). The plot was symmetrical, indicating no publication bias. This was confirmed by Egger’s test of the intercept (*p* = .83), which indicated no asymmetry of the funnel plot. The Duval and Tweedie trim and fill procedure indicated that no studies needed to be imputed.

#### Partner and infant outcomes

Unfortunately, it was not possible to synthesize outcomes with regard to partners since only three of the included studies included the partner in the interventions [41, 47, 49] and only two of these studies [47, 49] reported the partner’s distress outcomes. No effect of the intervention on paternal depression and anxiety was found in Feinberg and Kan [49], while women from the intervention group did report lower levels of distress than women from the control group (effect size .56 for depression; and .38 for anxiety). However, Daley-McCoy et al. [47] reported a significantly greater reduction in symptoms of depression for men (compared to the control group) in their psychoeducational intervention group aimed at the transition to parenthood, with a considerable effect size of .47. For women, no difference emerged between the intervention and the control group.

We were also unable to synthesize the effects of the interventions on the infant, as only three of the included studies assessed infant outcomes and the assessed outcomes showed too much variability. One of the studies measured the daily number of fetal movements (as a marker of maternal-fetal attachment [41];), one measured parent-child dysfunctional interaction and infant regulatory competence [49] and one measured birth outcomes (e.g. complications during delivery; mode of birth [43];). The results were mixed. Beattie et al. [43] did not find any effect on birth outcomes and the other two studies only for part of the measurements or for specific subgroups. More specifically, in the Feinberg and Kan [49] study, parents from the intervention group reported levels of dysfunctional interaction and distress in the relationship with their child around 6 months postpartum. Interestingly, the effect size for fathers (.70) was large (.34 for mothers). Furthermore, infants from the intervention group showed a longer duration of orienting (mother and father report aggregated) and greater soothability (father report only) at 6 months postpartum. With regard to fetal movements, Abkarzadeh et al. [41] reported that in both intervention groups (psychoeducation about attachment and a relaxation intervention), the number of counted movements increased (compared to the control group). This increase was only statistically significant for the educational attachment intervention group (and not for the relaxation group).

## Discussion

This meta-analysis focused on the effectiveness of preventive psychological interventions offered to universal populations of pregnant women on symptoms of depression, anxiety, and general stress. Paternal and infant outcomes were also included. The meta-analysis suggested that psychological interventions among pregnant women without a specific risk of psychopathology and implemented during pregnancy, are effective in the prevention of maternal distress symptomatology. The meta-analysis showed that these interventions have a moderate effect on the combined measure of distress (d = .52) as well as on depressive symptoms (d = .50), and stress (d = .52). The effect on anxiety (d = .30) was somewhat smaller. These results indicate that, next to indicated and selected prevention, universal prevention has value in its own right. Since the results with regard to anxiety and stress are based on a considerably lower number of studies, the effectiveness of universal prevention on the prevention of distress beyond depression should be interpreted with caution.

Two studies were outliers and thus excluded [48, 50]. These studies differed from the other studies in several respects. The Haga et al. [48] study was the only study in which a multimodal intervention was tested, consisting of elements of meta-cognitive therapy, mindfulness, acceptance and commitment therapy, positive psychology, cognitive-behavioral therapy, and psychoeducation. The online intervention consisted of 44 sessions. While these sessions took not much time to complete (about 10 min), the number of sessions was much higher than in the other studies. It is possible that the high number of sessions, but also the multimodal nature of the intervention, explain why this study found a much larger effect size than the other studies. It might be that by offering multiple elements of different interventions and therapies, women can choose those elements that work best for them to alleviate distress symptoms. The intervention in the Khorsandi et al. [50] study differed from the other interventions as it was exclusively focused on stress, namely psychoeducation about stress and how to handle signals of stress. The content seemed to be not exclusively geared to pregnant women, while this was the case for the other included interventions. Moreover, it was difficult to assess the methodological quality of this study. For example, no flow chart and no info on timing of measurements was reported. Also, lack of active and regular participation in the training stages of the intervention was described as an exclusion criterion. It is not clear if this criterion actually resulted in exclusion of participants, but if so, the sample could be biased towards more highly motivated women (potentially leading to a higher effect size). It is important to emphasize that exclusion of these two studies lead to more conservative effect estimates. As a result, the true effects might even be higher than the ones we reported.

The studies we included were aimed at universal prevention. The majority of the included studies (*n* = 9) excluded women with a diagnosis of anxiety or depression, or women who scored above a cut-off on questionnaires. The remaining three studies included all pregnant women (and their partners) regardless of pre-existing symptomatology or risk status, but did not exclude women with a diagnosis or (severe) symptoms. The overall mean baseline scores of all studies showed that the women had some symptoms, but that the depression, anxiety, and stress scores were well below clinical cut-off. While indicated and selected prevention efforts are exclusively aimed at women who are screened on their (considerable) level of distress symptomatology (using validated cut-off scores), or their relative risk on developing psychopathology based on the presence of one or more known risk factors (e.g. pregnancy complications, low social support), universal prevention targets those women who have no known risk factors and experience low to moderate levels of distress. The current analysis suggested a considerable effect on symptoms of depression, anxiety, or general stress for these women, indicating the added value of universal prevention of distress in pregnancy.

The included preventive interventions varied from (online) mindfulness-based self-help interventions to interventions consisting of multiple group sessions based on principles of established therapeutic techniques, such as cognitive-behavioural therapy (problem solving and communication skills) and interpersonal psychotherapy (underlining the importance of social relationships). Most interventions were exclusively aimed at pregnant women and included psychoeducation about postnatal distress, relaxation techniques and the acquisition of emotion regulation skills. Also, most interventions were offered in a group setting (in a local hospital) and facilitated by a mental health professional or a midwife. A minority of interventions was offered in a (internet-based) self-help format. A subset of interventions also offered postnatal sessions: these interventions were also aimed at the couple relationship and/or the transition to parenthood. We could not demonstrate that one type of intervention was more effective than the other types. However, due to the high heterogeneity among the interventions and the small number of studies per type of intervention, the analyses might have lacked sufficient power to demonstrate differences in effect.

When considering the three indicators of distress separately, we found considerable effect sizes for depression (*d* = .50), stress (*d* = .52), and a somewhat lower effect size for anxiety (*d* = .30). This implies that the interventions were effective on all three indicators of distress. Moreover, analyzing the indicators of distress separately resulted in less heterogeneity. It is important to keep in mind that most of the included studies focused on symptoms of depression (*n* = 10), and that the results for symptoms of stress and anxiety were based on a considerably lower number of studies (*n* = 5).

The impact of universal prevention on symptoms of *depression* is in line with conclusions of earlier reviews and meta-analyses showing the effectiveness of selected and indicated prevention for depression and depressive symptomatology [17, 19–21]. For example, one of the studies [51] in our meta-analysis considered the incidence of a depressive disorder by 6 weeks postpartum as an outcome. While 10 women from the control group were diagnosed with depression (9.3%), only three women (2.7%) were diagnosed in the intervention group. Other studies (*n* = 4) reported the percentage of women that scored above cut-off scores for depression, using the Edinburgh Postnatal Depression Scale (EPDS [38];). The rates varied between 11.8 and 40.6% for the control groups, and, in contrast, between 8.7 to 17.4% for the intervention groups. However, different cut-off scores were used (ranging from 10 to 14), which makes the percentages difficult to compare. To be able to detect whether universal prevention leads to less cases of a depressive disorder (and thus to genuinely assess the effect of universal prevention during pregnancy on the development of psychopathology), future studies are strongly encouraged to report the incidence rate of depression and other mental disorders as an outcome. When cut-off scores are used for this aim, it is important to use comparable cut-off score across studies.

While earlier reviews were not able to quantify the effect of universal prevention on symptoms of *anxiety* [21, 23], results of this meta-analysis indicate that, next to depression, universal prevention has a moderately preventing effect on symptoms of anxiety. As an accumulating number of studies indicated that women can experience considerable levels of anxiety symptomatology after childbirth [5, 8], even resulting in an anxiety disorder [13], it is an important finding that universal prevention apparently works to alleviate anxiety symptoms. However, the number of interventions focusing on the prevention of anxiety was rather low and the effect size seemed to be smaller (*d* = 0.30) than for depression (*d* = 0.50) and stress (*d* = 0.52). Therefore, in line with earlier meta-analyses [21, 23), we hope that future prevention trials will include anxiety as a target of intervention.

There are two other reviews, which examined effects on general *stress* [31, 32]. These reviews did not indicate an effect of antenatal universal prevention. However, these reviews included a mixture of universal, selective and indicated prevention, possibly explaining the different results. Also, there were differences in the nature of the stress measures included. In Fontein-Kuipers et al. [30] distress was broadly measured and included symptoms of depression and anxiety next to perception of stress, parenting stress, and parental worry. It is possible that different types of stress need different types of intervention, and that a potential effect of interventions on this broad index of stress would thus be more difficult to detect.

This meta-analysis showed that a minority of interventions focused on both partners. Only in three of the 12 included interventions, the partner was also involved. These interventions focused mainly on the functioning of the couple relationship during the transition to parenthood. Our meta-analysis showed that these interventions were less effective in preventing distress, than interventions that only included the mother. It might be that mothers with a partner willing to participate in a preventive intervention experience higher levels of social support at baseline, and therefore lower levels of distress. The intervention might thus be less effective for this group of women. However, given that both the content of the intervention (focus on the couple relationship), and the target group (couples) varied, no firm conclusion about the effectiveness of including the partner in preventive interventions can be drawn yet. Furthermore, preventing distress in partners might request a different approach, and it is thus worthwhile to also investigate interventions exclusively geared on the partner. Given the paucity of trials that focused on distress of the partner, and abundant research indicating that fathers also experience considerable postpartum distress [32, 34] which might also affect the child [36, 37], future trials should focus on the prevention of both maternal and paternal distress.

Likewise, we were not able to measure the effectiveness of the interventions on infant outcomes, as only three of the 12 included studies assessed (a variety of) infant outcomes. Beattie et al. [43] reported no effect on various birth outcomes of their mindfulness intervention. In Feinberg and Kan [49], parents (especially fathers) participating in a cognitive-behavioural based psychosocial prevention program reported lower levels of dysfunctional interaction and distress in the relationship with their child around 6 months postpartum. Also, infants from the intervention group showed a longer duration of orienting and greater soothability. Abkarzadeh et al. [41] reported that in both their intervention groups (psychoeducational attachment and relaxation), the number of counted fetal movements increased compared to the control group.

Given the well-established impact of parental distress on children’s well-being and development [9, 35, 52], future trials are encouraged to investigate whether the positive effects of the universally applied psychological interventions extend from mother to infant. Since parenting quality is a factor that can be modified by intervention [53], focusing on the inclusion of quality-related outcomes, such as soothability and parent-infant interaction [49] could be a promising pathway. For example, including observational measures of parental sensitivity for and responsiveness to stress signals of the infant could be included.

### Limitations

The current study has several limitations. First, most of the included studies focused on depressive symptomatology as an outcome. Therefore, we were unable to draw firm conclusions regarding the other indicators of distress, namely symptoms of anxiety and general stress. Second, because none of the included studies focused on child outcomes, no conclusions about the effectiveness of the interventions on infant well-being could be drawn. Third, only a limited number of studies included the partner, which means that the effectiveness of interventions during pregnancy on preventing distress of the partner could not be analyzed. Fourth, the risk of bias assessment indicated that a large part of studies was not sufficiently transparent in reporting all information necessary to give a quality judgement based on the Cochrane Risk of Bias Assessment tool. This was mainly a problem when judging the random sequence generation and the allocation procedure, in which respectively two-third and half of the studies did not report how they handled this. Also, judgement of the incomplete outcome data criterion revealed that almost half of the studies had to deal with relatively high drop-out rates and/or did not specify the reasons for drop-out adequately. However, subgroup analyses showed no association between overall methodological quality and the size of the effect. Fifth, to be able to detect whether universal prevention would make a difference in preventing distress (i.e. if universal prevention is worthwhile from a cost-effectiveness perspective) we compared the effect of universal prevention to routine care. While routine care can be provided by midwives, there were differences between studies as to which type of routine care women have access to during pregnancy. Also, not all studies provided sufficient details about what constituted regular care in their study. This means that the regular care condition might have varied between studies. To be able to detect if additional support during pregnancy could contribute to stress reduction among pregnant women compared to different types of routine care, future trials are recommended to provide details about regular care in their specific study setting.

## Conclusions

This meta-analysis suggests that universally applied psychological interventions during pregnancy are effective in preventing symptoms of maternal distress, at least with regard to depression While promising, the results with regard to anxiety and stress are based on a considerably lower number of studies, and therefore the effectiveness of universal prevention on the prevention of these types of distress should be interpreted with caution. However, the current meta-analysis offers sufficient indications that, beyond implementing preventive interventions tailored at at-risk women during pregnancy, prenatal services should be offered to all pregnant women, regardless of their risk status. Due to the mix of working elements in the included interventions, it seems too early to conclude what type of intervention should be offered. Importantly, since most studies focused on symptoms of depression, more research is necessary on the effectiveness of universal prevention on symptoms of anxiety and stress. Also, the partner should be included in future trials and, crucially, interventions should be designed and investigated that not only prevent maternal or paternal distress, but also prevent the negative effects of parental distress on the infant.

## Supplementary Information

**Additional file 1.**

## Data Availability

The extracted data is available upon request from the corresponding author.

## Abbreviations

AvS: Annemieke van Straten
CMA: Comprehensive Meta-analysis
CM: Charlotte Moyse
DSM-IV: Diagnostic and Statistical Manual of Mental Disorders
EPDS: Edinburgh Postnatal Depression Scale
HIV: human immunodeficiency virus
MC: Marketa Ciharova
MM: Marjolein Missler
NNT: Numbers-needed-to-be-treated
SES: Socioeconomic status; TD – Tara Donker
