# Supplementary material for: Universal prevention of distress aimed at pregnant women: a systematic review and meta-analysis of psychological interventions
Source: BMC Pregnancy Childbirth. 2021 Apr 1;21:276. doi: 10.1186/s12884-021-03752-2 (PMC8017784; doi:10.1186/s12884-021-03752-2)
Supplement: Supplementary file 1 — Additional file 1. [file 12884_2021_3752_MOESM1_ESM.docx]

**PubMed Session Results (15 Nov 2018)**

| **#** | **Query** | **Results** |
| --- | --- | --- |
| #5 | #1 AND #2 AND #3 AND #4 | 2,148 |
| #4 | randomized controlled trial[pt] OR controlled clinical trial[pt] OR randomized[tiab] OR randomised[tiab] OR placebo[tiab] OR drug therapy[sh] OR randomly[tiab] OR trial[tiab] OR groups[tiab] | 4,392,989 |
| #3 | "Stress, Psychological"[Mesh] OR "Anxiety"[Mesh:NoExp] OR "Depression"[Mesh] OR "Depressive Disorder"[Mesh:NoExp] OR "Depression, Postpartum"[Mesh] OR maternal distress[tiab] OR maternal stress[tiab] OR parenting distress[tiab] OR parenting stress[tiab] OR parental distress[tiab] OR parental stress[tiab] OR psychological distress[tiab] OR psychological stress[tiab] OR parental well-being[tiab] OR infant well-being[tiab] OR parental wellbeing[tiab] OR infant wellbeing[tiab] OR infant development[tiab] OR infant crying[tiab] OR infant sleeping[tiab] OR "Infant Health"[Mesh] OR infant health[tiab] OR baby health[tiab] OR newborn health[tiab] OR neonate health[tiab] OR neonatal health[tiab] | 336,914 |
| #2 | "Early Intervention (Education)"[Mesh] OR "Education"[Mesh] OR "Social Support"[Mesh] OR "Cognitive Therapy"[Mesh] OR "Preventive Health Services"[Mesh:NoExp] OR "Primary Prevention"[Mesh] OR "Health Education"[Mesh] OR "Health Promotion"[Mesh] OR early intervention*[tiab] OR social support[tiab] OR social network*[tiab] OR cognitive therap*[tiab] OR cognitive psychotherap*[tiab] OR primary prevention[tiab] OR health education[tiab] OR health promotion[tiab] | 1,042,708 |
| #1 | "Parents"[Mesh] OR "Pregnant Women"[Mesh] OR "Pregnancy"[Mesh] OR "Maternal Health Services"[Mesh] OR parent[tiab] OR parents[tiab] OR parental[tiab] OR father*[tiab] OR mother*[tiab] OR pregnan*[tiab] OR gravidit*[tiab] OR gestation*[tiab] OR placentat*[tiab] OR prepregnan*[tiab] OR conception*[tiab] OR preconception*[tiab] OR perinatal[tiab] OR prenatal[tiab] | 1,458,107 |

**Embase.com Session Results (15 Nov 2018)**

| **#** | **Query** | **Results** |
| --- | --- | --- |
| #5 | #1 AND #2 AND #3 AND #4 | 2,795 |
| #4 | random* OR factorial* OR crossover* OR cross NEXT/1 over* OR placebo* OR (doubl* AND blind*) OR (singl* AND blind*) OR assign* OR allocat* OR volunteer* OR 'crossover procedure'/exp OR 'double blind procedure'/exp OR 'randomized controlled trial'/exp OR 'single blind procedure'/exp | 2,314,538 |
| #3 | 'mental stress'/exp OR 'anxiety'/de OR 'depression'/de OR 'puerperal depression'/exp OR 'child health'/exp OR 'maternal distress':ab,ti,kw OR 'maternal stress':ab,ti,kw OR 'parenting distress':ab,ti,kw OR 'parenting stress':ab,ti,kw OR 'parental distress':ab,ti,kw OR 'parental stress':ab,ti,kw OR 'psychological distress':ab,ti,kw OR 'psychological stress':ab,ti,kw OR 'parental well-being':ab,ti,kw OR 'infant well-being':ab,ti,kw OR 'parental wellbeing':ab,ti,kw OR 'infant wellbeing':ab,ti,kw OR 'infant development':ab,ti,kw OR 'infant crying':ab,ti,kw OR 'infant sleeping':ab,ti,kw OR 'infant health':ab,ti,kw OR 'baby health':ab,ti,kw OR 'newborn health':ab,ti,kw OR 'neonate health':ab,ti,kw OR 'neonatal health':ab,ti,kw | 576,190 |
| #2 | 'early childhood intervention'/exp OR 'education'/exp OR 'social support'/exp OR 'cognitive therapy'/exp OR 'cognitive behavioral therapy'/exp OR 'preventive health service'/de OR 'primary prevention'/exp OR 'health education'/exp OR 'early intervention*':ab,ti,kw OR 'social support':ab,ti,kw OR 'social network*':ab,ti,kw OR 'cognitive therap*':ab,ti,kw OR 'cognitive psychotherap*':ab,ti,kw OR 'primary prevention':ab,ti,kw OR 'health education':ab,ti,kw OR 'health promotion':ab,ti,kw | 1,577,450 |
| #1 | 'parent'/exp OR 'pregnant woman'/exp OR 'pregnancy'/exp OR 'maternal health service'/exp OR parent:ab,ti,kw OR parents:ab,ti,kw OR parental:ab,ti,kw OR father*:ab,ti,kw OR mother*:ab,ti,kw OR pregnan*:ab,ti,kw OR gravidit*:ab,ti,kw OR gestation*:ab,ti,kw OR placentat*:ab,ti,kw OR prepregnan*:ab,ti,kw OR conception*:ab,ti,kw OR preconception*:ab,ti,kw OR perinatal:ab,ti,kw OR prenatal:ab,ti,kw | 1,638,562 |

**Ebsco / PsycINFO Session Results (15 Nov 2018)**

| **#** | **Query** | **Results** |
| --- | --- | --- |
| S5 | S1 AND S2 AND S3 AND S4 | 591 |
| S4 | DE "Treatment Effectiveness Evaluation" OR DE "Clinical Trials" OR DE "Mental Health Program Evaluation" OR DE "Placebo" OR TI placebo* OR AB placebo* OR AB randomly OR TX randomi* OR TI trial OR AB trial OR TX ((singl* OR doubl* OR trebl* OR tripl*) N3 (blind* OR mask* OR dummy)) OR TI (control* N3 (trial* OR study OR studies OR group*)) OR AB (control* N3 (trial* OR study OR studies OR group*)) OR TI factorial* OR AB factorial* OR TI allocat* OR AB allocat* OR TI assign* OR AB assign* OR TI volunteer* OR AB volunteer* OR TI (crossover* OR "cross over*") OR AB (crossover* OR "cross over*") OR TX (quasi N5 (experimental OR random*)) | 485,125 |
| S3 | DE "Psychological Stress" OR DE "Anxiety" OR DE "Depression (Emotion)" OR DE "Major Depression" OR DE "Postpartum Depression OR TI ("maternal distress" OR "maternal stress" OR "parenting distress" OR "parenting stress" OR "parental distress" OR "parental stress" OR "psychological distress" OR "psychological stress" OR "parental well-being" OR "infant well-being" OR "parental wellbeing" OR "infant wellbeing" OR "infant development" OR "infant crying" OR "infant sleeping" OR "infant health" OR "baby health" OR "newborn health" OR "neonate health" OR "neonatal health") OR AB ("maternal distress" OR "maternal stress" OR "parenting distress" OR "parenting stress" OR "parental distress" OR "parental stress" OR "psychological distress" OR "psychological stress" OR "parental well-being" OR "infant well-being" OR "parental wellbeing" OR "infant wellbeing" OR "infant development" OR "infant crying" OR "infant sleeping" OR "infant health" OR "baby health" OR "newborn health" OR "neonate health" OR "neonatal health") OR KW ("maternal distress" OR "maternal stress" OR "parenting distress" OR "parenting stress" OR "parental distress" OR "parental stress" OR "psychological distress" OR "psychological stress" OR "parental well-being" OR "infant well-being" OR "parental wellbeing" OR "infant wellbeing" OR "infant development" OR "infant crying" OR "infant sleeping" OR "infant health" OR "baby health" OR "newborn health" OR "neonate health" OR "neonatal health") | 202,014 |
| S2 | DE "Early Intervention" OR DE "Education" OR DE "Family Life Education" OR DE "Parent Training" OR DE "Social Support" OR DE "Cognitive Therapy" OR DE "Cognitive Behavior Therapy" OR DE "Primary Mental Health Prevention" OR DE "Health Education" OR DE "Health Promotion" OR TI ("early intervention*" OR "social support" OR "social network*" OR "cognitive therap*" OR "cognitive psychotherap*" OR "primary prevention" OR "health education" OR "health promotion") OR AB ("early intervention*" OR "social support" OR "social network*" OR "cognitive therap*" OR "cognitive psychotherap*" OR "primary prevention" OR "health education" OR "health promotion") OR KW ("early intervention*" OR "social support" OR "social network*" OR "cognitive therap*" OR "cognitive psychotherap*" OR "primary prevention" OR "health education" OR "health promotion") | 237,788 |
| S1 | DE "Parents" OR DE "Fathers" OR DE "Mothers" OR DE "Expectant Mothers" OR DE "Pregnancy" OR DE "Adolescent Pregnancy" OR TI (parent OR parents OR parental OR father* OR mother* OR pregnan* OR gravidit* OR gestation* OR placentat* OR prepregnan* OR conception* OR preconception* OR perinatal OR prenatal) OR AB (parent OR parents OR parental OR father* OR mother* OR pregnan* OR gravidit* OR gestation* OR placentat* OR prepregnan* OR conception* OR preconception* OR perinatal OR prenatal) OR KW (parent OR parents OR parental OR father* OR mother* OR pregnan* OR gravidit* OR gestation* OR placentat* OR prepregnan* OR conception* OR preconception* OR perinatal OR prenatal) | 419,806 |

**Ebsco / CINAHL Session Results (15 Nov 2018)**

| **#** | **Query** | **Results** |
| --- | --- | --- |
| S5 | S1 AND S2 AND S3 AND S4 | 815 |
| S4 | (MH "Clinical Trials+") OR (PT Clinical trial) OR (TX clini* N1 trial*) OR (TX ((singl* N1 blind*) or (singl* N1 mask*)) or TX ((doubl* N1 blind*) or (doubl* N1 mask*)) OR or TX ((tripl* N1 blind*) or (tripl* N1 mask*))) OR (TX randomi* control*) OR (MH "Random Assignment") OR ((TX random* allocat*) or (TX allocat* random*)) OR (TX placebo*) OR (TX (waitlist* or (wait* and list*)) and (control* or group))) OR ((TX "treatment as usual") or (TX tau)) OR (TX (control* N3 (trial* or study or studies or group*))) OR (MH "Quantitative Studies") | 552,344 |
| S3 | (MH "Stress, Psychological+") OR (MH "Anxiety") OR (MH "Depression") OR (MH "Depression, Postpartum") OR (MH "Child Health") OR TI ("maternal distress" OR "maternal stress" OR "parenting distress" OR "parenting stress" OR "parental distress" OR "parental stress" OR "psychological distress" OR "psychological stress" OR "parental well-being" OR "infant well-being" OR "parental wellbeing" OR "infant wellbeing" OR "infant development" OR "infant crying" OR "infant sleeping" OR "infant health" OR "baby health" OR "newborn health" OR "neonate health" OR "neonatal health") OR AB ("maternal distress" OR "maternal stress" OR "parenting distress" OR "parenting stress" OR "parental distress" OR "parental stress" OR "psychological distress" OR "psychological stress" OR "parental well-being" OR "infant well-being" OR "parental wellbeing" OR "infant wellbeing" OR "infant development" OR "infant crying" OR "infant sleeping" OR "infant health" OR "baby health" OR "newborn health" OR "neonate health" OR "neonatal health") OR KW ("maternal distress" OR "maternal stress" OR "parenting distress" OR "parenting stress" OR "parental distress" OR "parental stress" OR "psychological distress" OR "psychological stress" OR "parental well-being" OR "infant well-being" OR "parental wellbeing" OR "infant wellbeing" OR "infant development" OR "infant crying" OR "infant sleeping" OR "infant health" OR "baby health" OR "newborn health" OR "neonate health" OR "neonatal health") | 188,935 |
| S2 | (MH "Early Childhood Intervention") OR (MH "Education") OR (MH "Support, Psychosocial") OR (MH "Cognitive Therapy") OR (MH "Health Education") OR (MH "Health Promotion") OR TI ("early intervention*" OR "social support" OR "social network*" OR "cognitive therap*" OR "cognitive psychotherap*" OR "primary prevention" OR "health education" OR "health promotion") OR AB ("early intervention*" OR "social support" OR "social network*" OR "cognitive therap*" OR "cognitive psychotherap*" OR "primary prevention" OR "health education" OR "health promotion") OR KW ("early intervention*" OR "social support" OR "social network*" OR "cognitive therap*" OR "cognitive psychotherap*" OR "primary prevention" OR "health education" OR "health promotion") | 195,286 |
| S1 | (MH "Parents") OR (MH "Fathers") OR (MH "Mothers") OR (MH "Expectant Mothers") OR (MH "Pregnancy+") OR TI (parent OR parents OR parental OR father* OR mother* OR pregnan* OR gravidit* OR gestation* OR placentat* OR prepregnan* OR conception* OR preconception* OR perinatal OR prenatal) OR AB (parent OR parents OR parental OR father* OR mother* OR pregnan* OR gravidit* OR gestation* OR placentat* OR prepregnan* OR conception* OR preconception* OR perinatal OR prenatal) OR KW (parent OR parents OR parental OR father* OR mother* OR pregnan* OR gravidit* OR gestation* OR placentat* OR prepregnan* OR conception* OR preconception* OR perinatal OR prenatal) | 359,970 |

**Wiley / Cochrane Library Session Results (15 Nov 2018)**

| **#** | **Query** | **Results** |
| --- | --- | --- |
| #4 | #1 AND #2 AND #3 | 371 |
| #3 | ("maternal distress" OR "maternal stress" OR "parenting distress" OR "parenting stress" OR "parental distress" OR "parental stress" OR "psychological distress" OR "psychological stress" OR "parental well-being" OR "infant well-being" OR "parental wellbeing" OR "infant wellbeing" OR "infant development" OR "infant crying" OR "infant sleeping" OR "infant health" OR "baby health" OR "newborn health" OR "neonate health" OR "neonatal health"):ab,ti,kw | 4,570 |
| #2 | ((early NEXT intervention*) OR "social support" OR (social NEXT network*) OR (cognitive NEXT therap*) OR (cognitive NEXT psychotherap*) OR "primary prevention" OR "health education" OR "health promotion"):ab,ti,kw | 33,845 |
| #1 | (parent OR parents OR parental OR father* OR mother* OR pregnan* OR gravidit* OR gestation* OR placentat* OR prepregnan* OR conception* OR preconception* OR perinatal OR prenatal):ab,ti,kw | 70,948 |
